# Global Trends and Regional Variations in Studies of HIV/AIDS

**DOI:** 10.1038/s41598-017-04527-6

**Published:** 2017-06-23

**Authors:** Arash Baghaei Lakeh, Navid Ghaffarzadegan

**Affiliations:** Department of Industrial & Systems Engineering, Virginia Tech, Blacksburg, VA 24061 USA

## Abstract

We conduct textual analysis of a sample of more than 200,000 papers written on HIV/AIDS during the past three decades. Using the Latent Dirichlet Allocation method, we disentangle studies that address behavioral and social aspects from other studies and measure the trends of different topics as related to HIV/AIDS. We show that there is a regional variation in scientists’ approach to the problem of HIV/AIDS. Our results show that controlling for the economy, proximity to the HIV/AIDS problem correlates with the extent to which scientists look at the behavioral and social aspects of the disease rather than biomedical.

## Introduction

Since it was first detected in the early 1980s, AIDS has been studied by scientists around the world. Biomedical and epidemiological scientists have made substantial progress in understanding and controlling the disease over time. In the early years, HIV was discovered to be the cause of AIDS^1–3^. Later, scientists uncovered the modes of transmission and developed blood tests for diagnosing HIV^1–3^. By the early 1990s, the first antiviral drugs were developed to suppress HIV^1–3^. Together, these developments and efforts to educate vulnerable populations about the transmission mechanisms slowed down the progress of the disease during the mid-1990s, at least in the United States. Yet, it was also in the decade of the 1990s that the HIV/AIDS epidemic reached its peak in Sub-Saharan Africa^1^. Eventually, as a result of global and regional efforts to control the epidemic, both the HIV incidence and AIDS-related death rates have dropped after the year 2000^4^.

The complexity of the HIV/AIDS problem goes beyond biomedical aspects of the disease. Many challenges are behavioral and social, such as public awareness of the disease^5^, risk perception^6^, high-risk behaviors^7^, willingness to be tested^8^, social stigma^9^, and treatment adherence^10^. The infectious nature of the disease also adds to the social network complexity of the problem and to infection patterns^11^. Numerous multi-disciplinary studies have been conducted to uncover these socio-behavioral aspects of the disease.

From the early years, behavioral studies have shed light on the psychological factors corresponding to risky behaviors that can lead to HIV infection^12^. Along the same line of research, preventive behavioral studies have focused on reducing the chances of being infected by HIV and alleviating the adverse outcomes for patients with HIV^13^. These studies have examined how people can avoid risky behaviors by becoming more knowledgeable, motivated, and capable in the context of avoiding HIV infection^7^. HIV/AIDS is a disease with strong social stigma attached to it in many societies. Sociologists and anthropologists have contributed to HIV/AIDS research by proposing theories and models to understand and overcome this stigma^9^. Overall, the behavioral and social sciences (BSS) studies have helped in shaping a better understanding of the disease, recognizing people with the highest risk, designing preventive programs, and maintaining medical treatment for current patients.

Cumulatively, studies conducted by scientists within the fields of biomedical sciences and BSS have shaped our understanding of HIV/AIDS as a global problem. Despite this ostensible shared understanding, the knowledge production process orbiting around the problem of HIV/AIDS has happened incrementally over decades of research and within the context of scientific enterprise in many countries around the world. Yet, little is known about the trends of research and shifts in focus of researchers as related to the problem of HIV/AIDS. In this study, we investigate shifts in the scientific community’s focus on BSS aspects of HIV/AIDS through a textual analysis of more than 200,000 HIV/AIDS publications in the Scopus data set. Our goal is to evaluate global and regional trends and potential variations in research. One finding of our study is that, in the case of HIV/AIDS, the share of BSS research has been increasing over time and, controlling for the economy, geographical proximity to the problem is associated with more BSS studies.

## Data: HIV/AIDS Publications between 1985 and 2012

We constructed a dataset of all academic papers on the topic of HIV/AIDS as reported in the Scopus data set, totaling 264,102 papers. These papers were published on a variety of subjects related to HIV/AIDS during the period 1985 to 2012. Our criteria for inclusion of the papers in our dataset were existence of the words “HIV” or “AIDS” in the title or abstract of papers. For the analysis, we used the abstract of the papers, year of publication, and location of authors based on their institutional affiliations. Locations of authors were used to assign a paper to one or multiple countries. For example, a paper with authors from three different countries was assigned to those three countries. In the first stage, we excluded papers lacking an abstract, which reduced our dataset to a total of 209,608 papers.

**Figure 1:**
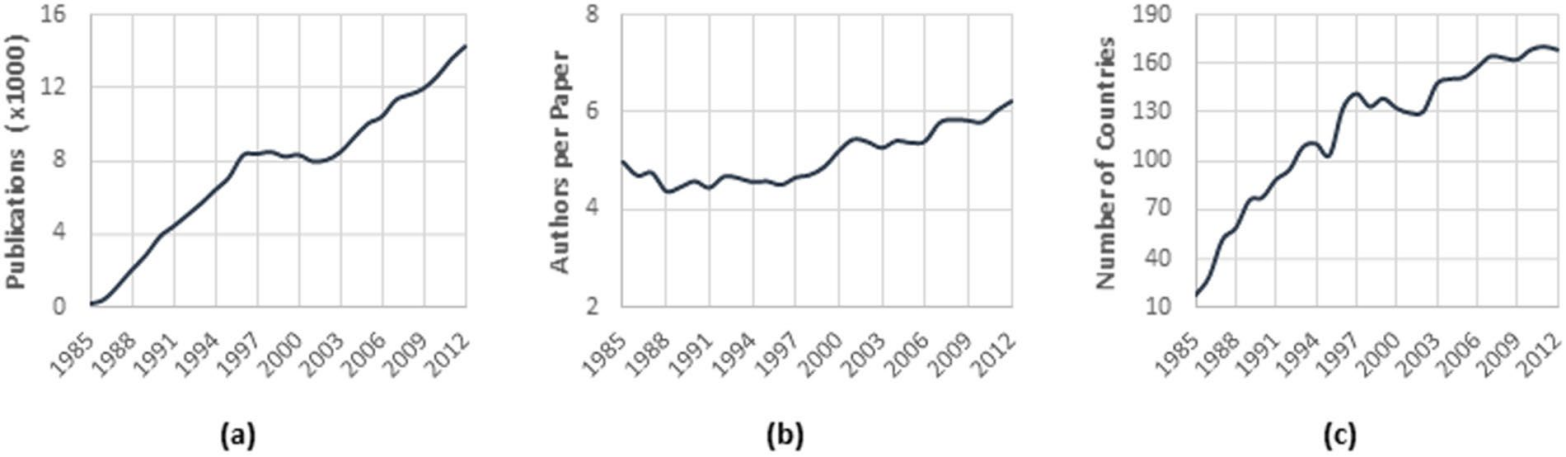
Trends in HIV/AIDS Publications: (**a**) global trend of publication; (**b**) average number of authors per paper; (**c**) number of countries contributing to HIV/AIDS research.

We first conducted a set of descriptive analysis at the aggregate level, as reported in Fig. 1. Figure 1a shows that the annual publication of scientific research on HIV/AIDS has been increasing since 1985 except for the period between 1996 and 2001. In 2012, the annual publication rate was about 14,250 papers per year, which shows almost 80% growth in a decade. Figure 1b reports collaboration patterns. The average number of authors per paper increased over time, indicating that research in the field has become more collaborative. In particular, the average number of authors per paper increased from about 4.2 in the 1980s and 1990s to about 6.2 authors per paper in 2012. Moreover, as Fig. 1c shows, more countries have been involved in these studies over time, and it is fair to say that the problem of HIV/AIDS has become a global field of research. As the figure shows, scientists in 168 countries had at least one publication on this topic in 2012.

## Increasing Focus on BSS Aspects of HIV/AIDS

**Figure 2:**
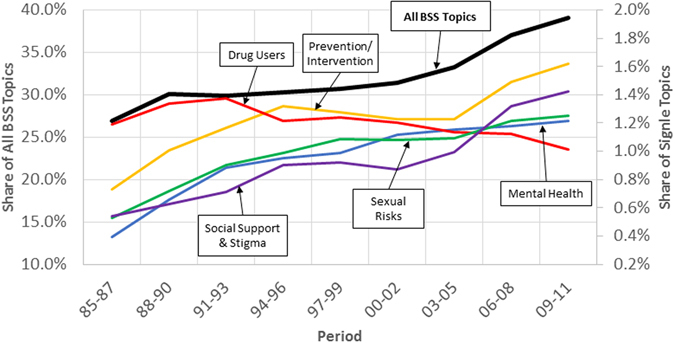
Share of aggregate BSS topics (left axis) and five individual topics (right axis) in HIV/AIDS research over time.

In the next step, we conducted textual analysis, looking at the abstract of HIV/AIDS publications over time. Methodologically, we implemented the Latent Dirichlet Allocation (LDA) on our data set. More information about LDA is provided in the method section; in short, in the LDA method, topics are identified based on the frequency of appearance and co-appearance of different words within all the documents. Our textual analysis of all paper abstracts shows an increasing trend in the share of BSS among HIV/AIDS publications (Fig. 2). In 2012, the share of BSS topics in HIV/AIDS research publications was more than 40%.

We also tracked the trends of individual BSS topics over time. As examples, Fig. 2 shows the share of five individual topics in HIV/AIDS research (i.e., Prevention/Intervention, Mental Health, Sexual Risks, Social Support & Stigma, and Drug Users); the remainder are reported in the SI. Some of these topics are gaining increasing attention in the scientific community. For example, the Prevention/Intervention, Mental Health, Sexual Risks, and Social Support & Stigma topics are all increasingly studied. Some other BSS topics, such as Drug Users show a declining trend in the share of publications in HIV/AIDS field since the beginning of the 21st century.

## Regional Variation in BSS Focus of HIV/AIDS Studies

**Figure 3:**
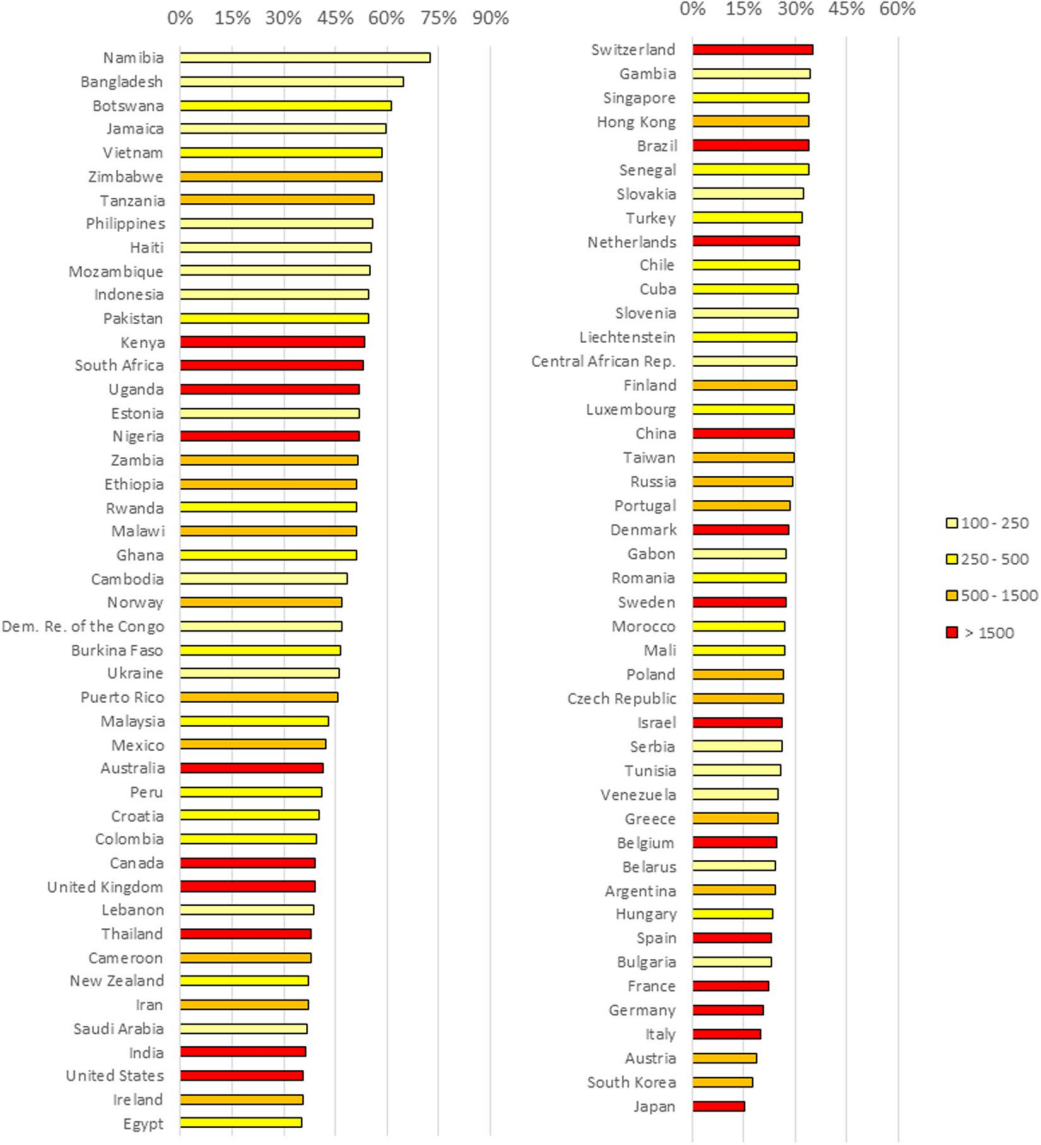
Share of BSS research in all HIV/AIDS papers of different countries. Note: For example, share of BSS research for Ethiopia is shown at 51% meaning that 51% of HIV/AIDS research content published by Ethiopian authors is on BSS topics. Only countries are shown that have published more than 100 papers during the period 1985 to 2012. Color coded by total number of HIV/AIDS papers.

Different countries have shown different level of focus on BSS in their HIV/AIDS research portfolio, as Fig. 3 illustrates. The share of BSS in HIV/AIDS publications from Namibia, Bangladesh, and Botswana is above 60%. Meanwhile, this proportion is less than 20% in Austria, South Korea, and Japan.

**Figure 4:**
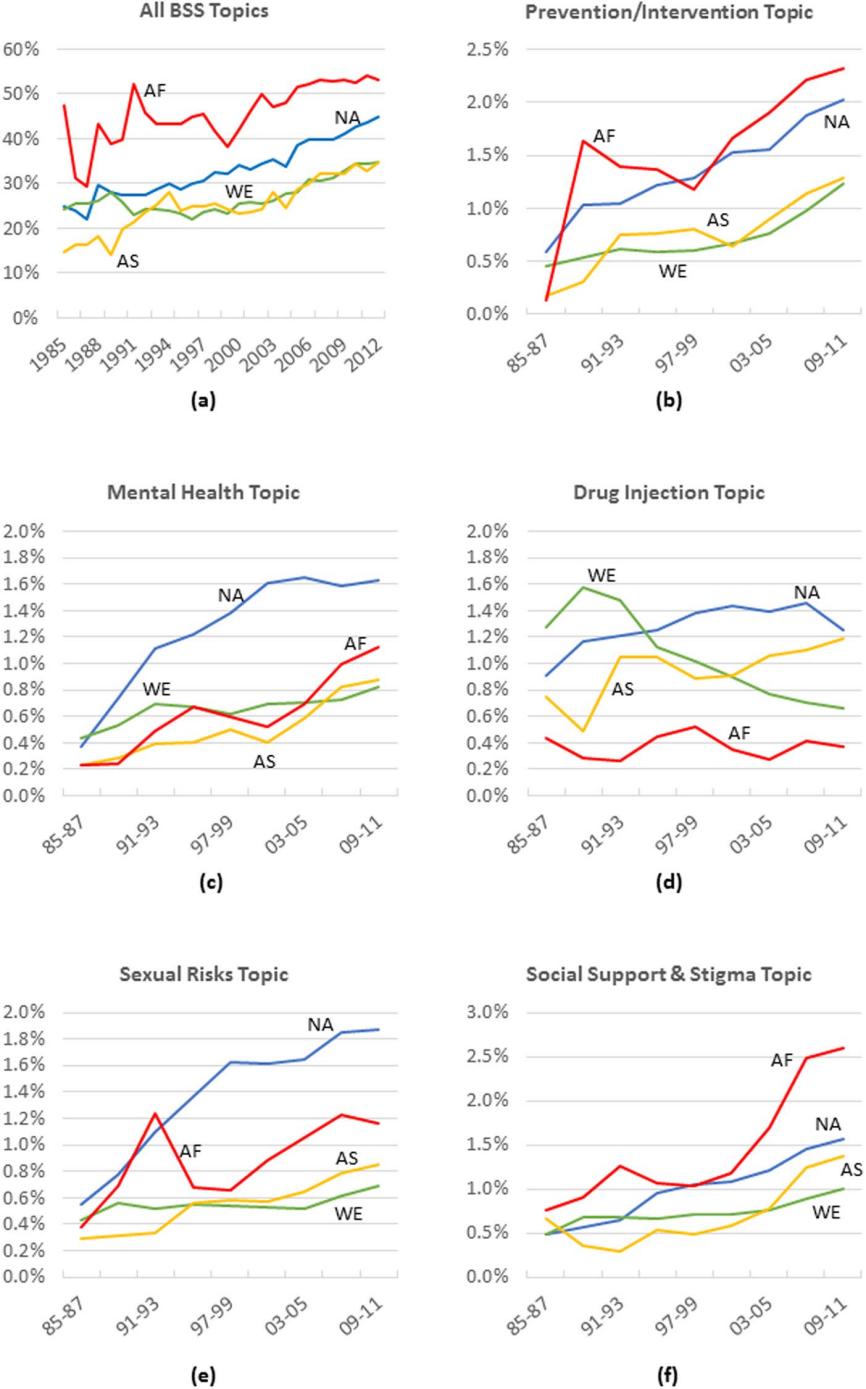
Regional variation in trends of BSS topics in the context of HIV/AIDS research. NA: North America, WE: Western Europe, AS: South and Southeast Asia, AF: Sub-Saharan Africa.

We observe a regional variation in BSS study trends, as Fig. 4 shows. Figure 4a shows varying regional trajectories in BSS studies of HIV/AIDS. In Sub-Saharan Africa, for instance, far more attention has been paid to BSS studies than elsewhere. The share of BSS studies among all HIV/AIDS research publications of Sub-Saharan African countries has been above 50% since 2005. In other regions, the share of BSS research has been increasing. The growth rate has been faster in South and Southeast Asia (with an average growth rate of 0.66% per year) and North America (with an average growth rate of 0.73% per year) compared with Western Europe (with an average growth rate of 0.34% per year).

Regional variation also exists among individual topics. Figure 4b–f demonstrate these variations for five sub-topics of BSS (i.e., Prevention/Intervention, Mental Health, Sexual Risks, Social Support & Stigma, and Drug Users). We observe that Sub-Saharan Africa and Northern America have shown more interest in Intervention/Prevention than Western Europe and South and Southeast Asia. On the topic of mental health and sexual risks as related to HIV/AIDS, the attention of North American scientists has been the highest in comparison to other regions. We observe that Western Europe showed a high interest in topic of Drug Users in the earlier years, but this interest has recently faded. All regions have shown an increasing interest in the Social Support &﻿ Stigma topic since 1985. The trend has been increasing at a higher rate for Sub-Saharan Africa.

Why does this variation between different regions in terms of their focus on the BSS aspects of HIV/AIDS exist? To study what drives BSS focus in HIV/AIDS research, we perform a panel data regression analysis. The factors we include in our analysis are the HIV/AIDS mortality rate and GDP per capita. We expect HIV/AIDS mortality to be associated with how researchers and science policy makers perceive the problem of HIV/AIDS. We represent the magnitude of the problem by HIV/AIDS mortality rate using the HIV mortality data from the “Global burden of disease study 2013” [Available from http://ghdx.healthdata.org/global-burden-disease-study-2013-gbd-2013-data-downloads]. Our BSS-related question is whether the higher rate of the problem is associated with higher BSS studies.

One other potential explanation for research focus is the research capacity or financial resources available for research. Biomedical research is more costly. By having more resources available for research, we expect that affluent countries be more capable of conducting biomedical research. This can affect the relative share of BSS in research portfolio of these countries. In our analysis, we control for GDP per capita as a proxy for countries’ economic prosperity.

**Figure 5:**
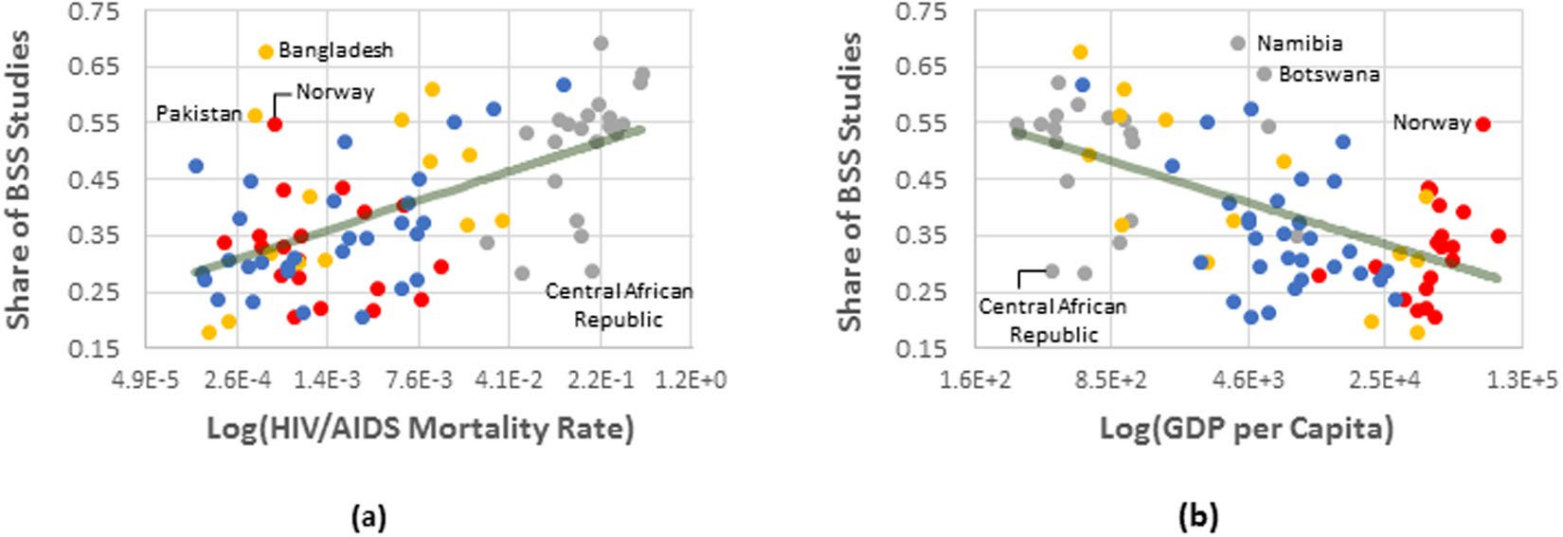
BSS focus of publications from different countries (average during the period 2006 to 2010) compared to their HIV/AIDS mortality rate in 2005 and GDP per capita (average during 2006 to 2010). Each dot corresponds to a country (Red: North America & Western Europe; Yellow: Southern & Southeast Asia; Gray: Sub-Saharan Africa; Blue: Rest of the world). The solid lines show the correlation lines. Countries that are two standard deviation further from the correlation line are labeled.

Our regression analysis shows there is a positive association between countries’ HIV/AIDS mortality rate and the ratio of BSS topics in their research publications on HIV/AIDS (p < 0.001). The detailed results are reported in the methods section. For the sake of simplicity, Fig. 5 depicts the correlation (*ρ* = 0.58 for relationship between percentage of BSS and HIV/AIDS mortality rate and *ρ* = −0.56 for relationship between percentage of BSS and GDP per capita). Our analysis also provides evidence that supports a negative correlation between GDP per capita in countries and the ratio of BSS topics in their publications (p < 0.001).

## Discussion

By constructing and analyzing a large data set of more than 200,000 papers, and by implementing a statistical natural language processing method (Latent Dirichlet Allocation), we were able to investigate the trends of studies in HIV/AIDS research in different regions. We found interesting trends in behavioral and social studies of HIV/AIDS. We observe that there is variation among different countries in terms of their focus on HIV/AIDS. The BSS focus has, on average, been the highest in Sub-Saharan Africa. Furthermore, we find an association between HIV/AIDS mortality and the percentage of BSS topics in HIV/AIDS research.

If we assume scientists’ responses to a problem are informed by the nature of the problem they observe in their proximity, our findings will have several implications. The scientific community’s focus on the “same” medical disease may vary across different regions and times, and this could speak to the varying nature of a seemingly similar disease in different regions and different time periods. If one assumes that a society’s understanding of a problem materializes in the form of scientific publications, our findings imply that the problem of HIV/AIDS is conceived to be more behavioral and social in places with higher mortality rates. The takeaway from this finding for science policy makers is that in defining priorities of research for a specific problem, the problem should not be looked at from an isolated perspective. To the contrary: it is of the utmost importance to nail down the problem in the context of each country and set priorities based on that perspective.

Our findings can be seen in the light of several prior science policy studies. The concept of “reconciling supply of science with societies’ demand”^14, 15^ implies that scientists’ approach to study a disease can partially reflect societal demand. Under this concept, a higher focus on the BSS aspects of HIV/AIDS in a country implies that those aspects of HIV/AIDS are perceived to be more critical in that region. Such premise seems reasonable: the broadly adverse social, cultural, and economic effects of HIV/AIDS on regions with high prevalence has made it a challenging epidemic to be tackled in those regions^16^. On the other hand, in low prevalence countries, more focus on biomedical studies may reflect the different nature of the same disease in those regions. In countries with less significant health challenges we often observe higher literacy, better quality of life, and more awareness about health. Consequently, less stress is placed on behavioral and social interventions than biomedical solutions. This premise is in line with medicalization and biomedicalization of health as studied by sociologists of health (e.g., ref. 17). We have also shown a negative association between GDP per capita and the ratio of BSS research in the contest of HIV/AIDS. This result is in line with previous studies that have shown affluent countries publish more biomedical papers^18^. Furthermore, our findings resonate with recent arguments on significant contributions of behavioral and social sciences to health^19^.

We focused on journal publications, but we admit that they are not the ideal representation of all research activities around a topic. Some research studies may not turn to journal publications and some may fail to get published. Different journals and different fields also have different standards and norms for publications. We also acknowledge that GDP per capita is not an accurate proxy for research spending of different countries. However, as used in other past science policy studies (e.g., ref. 18), GDP is a well-established measure for economy with relatively accurate data. Many other measures (such as research spending) are inconsistently calculated for different countries and, moreover, are not available for all countries. We think these limitations can be addressed in other future studies. Our study was a first major step and benefited from incorporating data analytics to uncover behavioral and social science contributions to health. We report details of our constructed data set in SI.

## Methods

### Topic Modeling

Different techniques can be used to extract the BSS theme within publications on HIV/AIDS. Simple approaches include inference solely based on keywords and authors’ departmental affiliations. These are easy to implement but have limitations. Assuming keywords are a sufficient description of content, and that different keywords have the same weight in describing an entire paper, is too simplistic. Departmental affiliation is not necessarily an accurate indicator of an author’s research; for example, faculty members in a public health department may do different types of research and address different topics. Furthermore, the relative weight of each keyword or contribution of each author in a given publication is unclear; is a paper with three authors, each one affiliated with three different departments of psychology, medicine, and public health, a BSS paper? If so, what topic within BSS? One needs to go deeper into the content of the paper to answer these questions.

Advancements in the data sciences help us conduct a more precise analysis. We address this methodological issue by using a Bayesian statistical technique known as Latent Dirichlet Allocation (LDA) that helps find the latent topics that generate a set of documents^20^. The underlying assumption of this method is that documents consist of probability distributions over a set of topics, while topics are probability distributions over the set of unique words (or corpus) that generate the entire set of documents. The output of an LDA implementation over a set of documents includes two pieces: a probability distribution over topics for each of the documents and a probability distribution over all unique words within the data set for each of the topics. The first output helps us know which topics are generating a given document and the second output helps us define each of the topics. LDA has been applied in a wide range of studies, including science and innovation studies^21–24^. For more information on the method, see ref. 20.

In this paper, we use the abstracts of papers as the set of documents to implement LDA. After removing the common words in English by using a standard stop list, we generated the corpus of unique words that generates all abstracts in our data set. The size of this corpus was 17,783,690 unique words. In our analysis we limited the set of unique words only to the ones that have been repeated in at least 100 abstracts. By applying this rule, the size of our corpus was reduced to 11,192 unique words. To implement LDA, three parameters need to be specified: the number of topics, a topic smoothing coefficient, and a term smoothing coefficient. To have a model with a better fit, using large number of topics is recommended. This, however, poses the problem of confronting topics that are indistinguishable in a meaningful way for human understanding^25^. In some prior topic modeling applications in the context of science and innovation^21, 22^, researchers have chosen to use 100 topics for their analysis. Following their lead, we also chose 100 topics to implement LDA in our data set. For topic and term smoothing parameters, 0.1 has typically been chosen as the default value^21, 22^. However, since we are looking for more finely separated topics and since the topic space of our documents is narrower (all papers are about HIV/AIDS), we reduced the term smoothing parameter to 0.01. This helps us have more discrete topics^21^. We used the python LDA 1.0.4 package to perform our analysis [Available at https://pypi.python.org/pypi/lda.].

Many of the topics generated by the LDA do not necessarily show a discipline or field of research. In fact, each abstract is a mixture of all the topics with different probabilities. However, we expect that, on average, topics belonging to BSS studies will have higher probabilities assigned to them in BSS publications. Based on this assumption, we anticipate that pairs of topics related to a specific body of knowledge are more likely to have co-occurrence probability (defined as the multiplication of the individual probabilities) in a publication compared with a pair of topics each related to a different fields of study. By making this assumption, we can look at the co-occurrence network of the topics, as uncovered by LDA, to find fields of research within our publication data set.

We generate the topic network by considering the five most probable topics of each document. Each of these five have, on average, a probability higher than 5%. Moreover, their average cumulative probability is greater than 70%. In our topic network, each node represents a topic. Topics are connected to each other based on their co-occurrence probability in all papers. The Louvain clustering^26^ was implemented on this network to find the communities of topics corresponding to fields of research. Three clusters were found. Cluster 1 includes topics related to medical case studies; Cluster 2 includes BSS topics; and cluster 3 includes biomedical topics. The individual topics and these clusters are reported in the SI. The robustness of these clusters against variability of input data was confirmed through sensitivity analysis for random sub-samples of papers. Results are presented in the SI. As another robustness check, we also generated a network with the eleven most probable topics of each paper. Each of these eleven topics, on average, have probabilities higher than 1%. Performing Louvain clustering on this network provided us with very similar clusters.

Finally, we would like to mention that a benefit of using LDA is to count for multidisciplinary works. Since in this method a topic of a paper is a probability distribution over different potential topics, a paper can be considered as x% BSS and y% biomedical. These percentages are estimated based on the words used in the abstracts.

### Panel Regression Analysis

To investigate the variation among different countries in terms of their focus on BSS aspects of HIV/AIDS, we performed a panel data regression analysis. As mentioned earlier, we had access to publication data for the period 1985 to 2012. The HIV/AIDS mortality rate estimates are available for the years 1990, 1995, 2000, 2005, 2010, and 2013. In our analysis, we regressed the average share of BSS topics during years *i* + *1* to *i* + *5* on the HIV/AIDS mortality rate of countries in year *i* controlling for their average GDP per capita during those years. Overall, our analysis includes 84 panels (countries with more than 100 papers on the topic HIV/AIDS during the period 1985-2012) for which we have four observations (*i* took the values 1990, 1995, 2000, and 2005).

Table 1 reports the summary of our regression analysis. We used a pooled least squares regression with Driscoll-Kraay standard errors^27, 28^ in our main model (Model 1). Due to the nature of our problem, we are concerned about heteroscedasticity and autocorrelation in our regression analysis. However, the selected model is robust against heteroskedasticity and autocorrelation. Other potential models such as Huber-White and Newey-West have also been used to confirm our regression results (see the results in SI). Due to the small number of observations per panel, we did not use a fixed effect model in our analysis, as we did not have enough variation. We tested for multicollinearity in our data and did not find an evidence for a potential problem (un-centered VIF: 1).

**Table 1:** Panel data regression results for percentage of BSS topics.

		Dependent Variable: Percentage of BSS topics in HIV/AIDS Research	
		Model 1	Model 2
**Independent Variables**	Log(HIV/AIDS Mortality)	0.016** (0.003)	0.014** (0.002)
	Log(GDP per Capita)	−0.027** (0.005)	−0.023** (0.003)
	Intercept	0.679** (0.009)	0.606** (0.026)
	*F*(2, 83)	12042.55***	
	*R* ^2^	0.2807	
	*Wald χ* ^2^		172.17***

*p < 0.05 **p < 0.001 ***p < 0.0001. Standard errors are presented in parentheses.

As a robustness check for our model selection, we performed the same analysis in Model 2 with another approach. In Model 2, we used a generalized least square model with heteroskedastic panels and first order autoregression. As Table 1 shows, the results from models 1 and 2 are very similar. However, since it is recommended to use the generalized least square model when the number of observations per panel is larger than the number of panels^21^, we selected Model 1 as our primary model in this paper. Overall, our models offer consistent results showing a positive association between HIV/AIDS mortality rate of countries and their focus on BSS aspects of HIV/AIDS. Our models also provide evidence to support a negative association between GDP per capita of countries and share of BSS in their HIV/AIDS publications.

## Electronic supplementary material

Appendix

Dataset
