# Supplementary material for: Global Trends and Regional Variations in Studies of HIV/AIDS
Source: Sci Rep. 2017 Jun 23;7:4170. doi: 10.1038/s41598-017-04527-6 (PMC5482881; doi:10.1038/s41598-017-04527-6)
Supplement: Supplementary file 1 — Appendix [file 41598_2017_4527_MOESM1_ESM.pdf]

Global Trends and Regional Variations in Studies of HIV/AIDS

Arash Baghaei Lakeh and Navid Ghaffarzadegan*

Department of Industrial & Systems Engineering, Virginia Tech

*correspondence to navidg@vt.edu

Supplementary Information

LDA Topics

In Table S1, we report the topics from our topic modeling analysis. For each topic we report the top 20 most probable words. The clusters in which each topic belongs to is also reported.

Table S1.
The detailed information of LDA topics.

Topic	Top 5 Cluster	Top 11 Cluster	Top 20 Words
**1**	2	2	support, living, people, social, family, hiv, stigma, hiv/aids, study, disclosure, care, status, members, families, aids, hiv-positive, caregivers, health, discrimination, work
**2**	1	1	oral, malaria, diarrhea, infection, intestinal, hiv, gastrointestinal, lesions, candidiasis, infections, mucosal, diarrhoea, associated, chronic, saliva, parasites, common, sp, stool, cryptosporidium
**3**	2	2	cost, costs, hiv, estimated, treatment, life, number, impact, cost-effectiveness, model, data, year, analysis, total, methods, coverage, effectiveness, compared, health, estimates
**4**	2	2	health, hiv/aids, aids, countries, public, policy, development, global, epidemic, international, national, government, people, access, economic, developing, social, policies, prevention, rights
**5**	1	1	patients, hiv-infected, study, hiv-positive, patient, hiv, methods, compared, clinical, infected, conclusions, higher, conclusion, treated, objective, background, included, hiv-negative, performed, aim
**6**	3	3	cells, cell, nk, dendritic, dc, expression, surface, molecules, dcs, cd4, receptor, human, adhesion, role, receptors, hiv, expressed, target, dc-sign, complement
**7**	2	2	care, health, services, medical, providers, service, primary, access, treatment, hiv, management, quality, system, clinics, patient, facilities, clinic, healthcare, provide, public
**8**	2	2	mortality, disease, diseases, deaths, countries, death, health, morbidity, infectious, developing, infections, major, population, people, burden, hiv/aids, worldwide, life, problem, rates

**9**	3	3	cells, cd4+, cell, lymphocytes, t-cell, cd8+, cd4, cd8, blood, expression, activation, peripheral, lymphocyte, immune, memory, subsets, flow, individuals, infection, increased
**10**	3	3	ccr5, cxcr4, receptor, entry, hiv-1, chemokine, receptors, coreceptor, infection, cells, cd4, r5, cell, chemokines, x4, strains, rantes, viral, human, virus
**11**	2	2	exposure, risk, hiv, transmission, workers, infection, health, occupational, care, blood, dental, exposures, hepatitis, control, medical, procedures, injuries, prophylaxis, exposed, pep
**12**	3	3	mice, cells, human, bone, marrow, vivo, cell, model, mouse, vitro, stem, hematopoietic, growth, murine, transgenic, normal, rats, studies, days, transplantation
**13**	2	2	sexual, adolescents, young, youth, sexually, health, adolescent, reproductive, sex, family, contraceptive, transmitted, years, pregnancy, risk, girls, age, contraception, intercourse, education
**14**	3	3	data, analysis, set, method, methods, based, model, study, models, prediction, developed, correlation, algorithm, validity, approach, predictive, predicted, system, test, reliability
**15**	1	1	plasma, concentrations, dose, drug, concentration, ritonavir, pharmacokinetic, pharmacokinetics, study, oral, administration, daily, days, indinavir, auc, clearance, doses, day, saquinavir, exposure
**16**	3	3	development, review, therapeutic, potential, novel, viral, target, understanding, agents, molecular, targets, approaches, drugs, drug, discovery, design, strategies, approach, including, biological
**17**	1	1	ci, risk, cohort, incidence, mortality, survival, follow-up, months, death, interval, confidence, associated, rate, study, years, ratio, hazard, time, compared, rates
**18**	2	2	ci, associated, factors, risk, odds, analysis, regression, study, ratio, logistic, multivariate, confidence, age, interval, association, aor, methods, adjusted, conclusions, prevalence
**19**	1	1	aids, disease, progression, hiv, infection, time, early, diagnosis, clinical, years, individuals, survival, course, late, associated, rapid, death, stage, long-term, infected
**20**	1	1	levels, serum, higher, level, plasma, concentrations, lower, increased, compared, correlated, high, elevated, low, measured, correlation, controls, concentration, values, normal, markers
**21**	2	2	increased, increase, time, changes, number, period, rate, decreased, decrease, change, observed, rates, incidence, decline, increasing, increases, year, trends, proportion, remained
**22**	1	1	patients, hospital, study, cases, years, age, medical, period, clinical, diagnosis, retrospective, january, objective, total, clinic, methods, records, university, diagnosed, included
**23**	1	1	renal, disease, patients, risk, kidney, hiv, failure, hypertension, cardiovascular, heart, function, cardiac, associated, chronic, hiv-infected, dysfunction, transplantation, diabetes, factors, transplant

**24**	1	2	children, hiv-infected, age, months, infected, years, child, infants, adults, pediatric, hiv, mothers, born, uninfected, infection, paediatric, adult, childhood, life, study
**25**	3	3	cells, virus, hiv-1, cell, infected, replication, viral, infection, human, blood, peripheral, mononuclear, culture, pbmc, primary, lines, macrophages, immunodeficiency, cultures, lymphocytes
**26**	1	1	patients, cmv, infections, pneumonia, pcp, infection, prophylaxis, opportunistic, carinii, pneumocystis, cytomegalovirus, aids, herpes, toxoplasmosis, retinitis, disease, hiv-infected, bacterial, respiratory, zoster
**27**	2	2	hiv, rights, reserved, article, review, published, discussed, current, clinical, treatment, issues, presented, university, literature, society, press, management, paper, science, elsevier
**28**	3	3	activity, inhibition, antiviral, inhibited, inhibitory, replication, vitro, inhibit, hiv-1, anti-hiv, potent, activities, cells, concentrations, compounds, virus, human, effects, concentration, g/ml
**29**	3	3	production, ifn-, expression, cytokines, cytokine, cells, tnf-, factor, il-2, levels, increased, ifn, response, tnf, monocytes, il-6, necrosis, macrophages, il-10, mrna
**30**	1	1	cd4, count, cell, counts, cd4+, lt, patients, lymphocyte, cells/, median, gt, cells/mm3, hiv-infected, clinical, hiv, t-cell, baseline, low, cells/mm, lower
**31**	2	2	syphilis, transmitted, sexually, genital, std, infection, women, infections, sti, hiv, stds, hsv-2, herpes, transmission, diseases, stis, prevalence, chlamydia, semen, vaginal
**32**	1	1	lymphoma, ks, kaposi, sarcoma, ebv, cases, patients, hhv-8, lymphomas, nhl, disease, associated, chemotherapy, non-hodgkin, tumor, b-cell, primary, virus, tumors, herpesvirus
**33**	2	2	medical, physicians, guidelines, patient, ethical, practice, issues, general, problems, health, informed, recommendations, decision, care, legal, consent, medicine, physician, public, questions
**34**	3	3	resistance, mutations, drug, mutation, hiv-1, rt, resistant, virus, susceptibility, variants, genotypic, viruses, mutant, associated, wild-type, strains, reverse, isolates, drug-resistant, phenotypic
**35**	2	2	women, alcohol, states, african, black, hiv, american, united, percent, reported, white, risk, rates, abuse, substance, persons, data, differences, population, hispanic
**36**	1	1	fat, patients, metabolic, lipodystrophy, insulin, cholesterol, associated, hiv-infected, lipid, glucose, increased, changes, body, mitochondrial, antiretroviral, lipoatrophy, therapy, syndrome, levels, resistance
**37**	1	1	zidovudine, azt, combination, lamivudine, zdv, tenofovir, therapy, nucleoside, didanosine, treatment, stavudine, ddi, drug, 3tc, abacavir, drugs, d4t, toxicity, antiretroviral, tdf
**38**	2	2	intervention, participants, study, baseline, group, follow-up, months, trial, design, control, hiv, program, interventions, randomized, measures, outcome, compared, conclusions, objective, methods

**39**	2	2	africa, south, rural, african, sub-saharan, hiv, prevalence, areas, countries, uganda, urban, high, kenya, epidemic, region, district, tanzania, southern, study, population
**40**	3	3	membrane, fusion, cell, cells, protein, proteins, peptide, surface, uptake, transport, membranes, lipid, peptides, cellular, plasma, microscopy, intracellular, delivery, particles, viral
**41**	3	3	gag, protein, rna, virus, rev, hiv-1, viral, proteins, domain, type, immunodeficiency, particles, human, assembly, region, processing, sequence, mutant, cleavage, amino
**42**	2	2	prevalence, years, hiv, age, population, data, older, males, higher, aged, rates, females, infection, adults, women, general, seroprevalence, incidence, rate, study
**43**	2	2	risk, sexual, behavior, hiv, behaviors, factors, interventions, prevention, condom, behavioral, perceived, associated, social, risky, sex, change, relationship, self-efficacy, study, reduction
**44**	1	1	group, subjects, groups, compared, control, controls, study, higher, hiv-positive, hiv+, differences, difference, individuals, versus, lower, hiv-negative, healthy, hiv-, test, statistically
**45**	3	3	binding, structure, residues, protein, site, peptide, hiv-1, structures, interactions, structural, complex, molecular, interaction, protease, affinity, complexes, conformation, crystal, amino, conformational
**46**	2	2	hiv, trials, clinical, vaccine, phase, trial, efficacy, effective, vaginal, development, studies, transmission, potential, prevention, microbicide, prevent, microbicides, safety, protection, gel
**47**	1	1	patients, hiv, aids, infection, seropositive, asymptomatic, subjects, stage, disease, clinical, hiv-seropositive, seronegative, stages, cdc, symptomatic, individuals, control, hiv-seronegative, early, group
**48**	1	1	haart, therapy, antiretroviral, active, highly, patients, hiv-infected, hiv, treatment, receiving, era, initiation, individuals, introduction, immune, treated, reconstitution, associated, anti-retroviral, decreased
**49**	1	1	treatment, therapy, drug, drugs, antiretroviral, clinical, effects, agents, therapeutic, combination, effective, hiv, therapies, management, regimens, efficacy, resistance, side, interactions, treatments
**50**	1	1	brain, csf, cns, system, central, nervous, dementia, neurological, fluid, cerebrospinal, pml, astrocytes, aids, encephalitis, disease, neuronal, microglia, hiv, disorders, cerebral
**51**	2	2	studies, data, review, trials, evidence, literature, published, criteria, identified, included, search, articles, outcomes, systematic, methods, clinical, conclusions, interventions, controlled, reports
**52**	1	1	weeks, patients, study, adverse, week, trial, treatment, events, randomized, placebo, group, daily, baseline, efficacy, safety, regimen, arm, subjects, received, nvp
**53**	3	3	compounds, activity, derivatives, synthesized, acid, anti-hiv, inhibitors, synthesis, series, compound, potent, hiv-1, analogues, novel, antiviral, active, activities, prepared, group, rights

**54**	3	3	assay, sensitivity, detection, assays, samples, specificity, test, method, methods, tests, laboratory, diagnostic, sensitive, clinical, rapid, standard, performance, testing, diagnosis, laboratories
**55**	1	1	hcv, hepatitis, hbv, virus, liver, infection, patients, chronic, coinfection, hiv, coinfected, hbsag, co-infected, anti-hcv, genotype, co-infection, fibrosis, cirrhosis, ribavirin, prevalence
**56**	1	1	tuberculosis, tb, cases, treatment, patients, pulmonary, mycobacterium, control, infection, high, active, disease, isoniazid, hiv, diagnosis, positive, sputum, tuberculin, case, culture
**57**	1	1	protease, inhibitors, inhibitor, reverse, transcriptase, pi, antiretroviral, nucleoside, drugs, pis, nnrti, therapy, regimens, drug, non-nucleoside, patients, combination, indinavir, treatment, ritonavir
**58**	1	1	viral, load, rna, plasma, copies/ml, patients, therapy, levels, virological, baseline, antiretroviral, suppression, response, vl, failure, virologic, undetectable, loads, median, log
**59**	2	2	model, data, models, time, method, estimates, population, dynamics, methods, based, approach, estimate, parameters, paper, distribution, analysis, proposed, mathematical, number, rate
**60**	3	3	gp120, envelope, antibodies, hiv-1, binding, gp41, peptide, neutralizing, v3, glycoprotein, antibody, peptides, cd4, env, neutralization, region, virus, human, epitope, epitopes
**61**	3	3	subtype, hiv-1, sequences, subtypes, sequence, strains, genetic, analysis, isolates, viruses, region, env, diversity, regions, variants, phylogenetic, gene, recombinant, identified, samples
**62**	3	3	immune, infection, viral, host, system, response, hiv, role, mechanisms, infections, immunity, virus, disease, replication, responses, pathogenesis, factors, cellular, chronic, understanding
**63**	1	1	cancer, women, hpv, cervical, anal, human, infection, lesions, carcinoma, risk, hiv-positive, types, cancers, squamous, papillomavirus, screening, intraepithelial, hiv-infected, hiv, neoplasia
**64**	2	2	social, hiv/aids, study, qualitative, interviews, paper, women, context, analysis, health, focus, article, cultural, people, gender, understanding, experiences, issues, relationships, findings
**65**	1	1	patient, case, report, diagnosis, presented, man, reported, cases, infection, lesions, rare, revealed, treatment, developed, clinical, hiv, acute, fever, history, symptoms
**66**	3	3	method, ph, conditions, chromatography, mass, concentration, gel, acid, human, analysis, liquid, mm, water, phase, temperature, developed, solution, high, range, detection
**67**	3	3	dna, pcr, chain, reaction, polymerase, samples, detected, gene, analysis, detection, blood, genotype, amplification, proviral, study, individuals, polymorphisms, allele, sequences, presence
**68**	2	2	drug, users, injection, idus, injecting, hiv, risk, drugs, intravenous, treatment, idu, abuse, cocaine, methadone, heroin, sharing, needle, prison, syringe, syringes

**69**	1	1	patients, pulmonary, diagnosis, lung, tuberculosis, clinical, infection, disease, cases, mycobacterium, chest, diagnostic, positive, respiratory, mac, culture, sputum, avium, findings, symptoms
**70**	1	1	weight, body, loss, vitamin, mass, nutritional, growth, food, hiv-infected, status, intake, deficiency, wasting, supplementation, bone, low, increased, associated, bmi, muscle
**71**	2	2	prevention, program, health, community, programs, interventions, project, hiv, education, evaluation, training, activities, implementation, national, intervention, hiv/aids, effective, development, support, programme
**72**	2	2	men, sex, sexual, msm, hiv, partners, risk, gay, anal, homosexual, reported, intercourse, unprotected, male, heterosexual, bisexual, circumcision, hiv-positive, partner, behavior
**73**	3	3	dna, rt, reverse, rna, hiv-1, transcriptase, integration, enzyme, integrase, activity, rnase, polymerase, strand, site, viral, synthesis, primer, transcription, sequence, transfer
**74**	3	3	nef, hiv-1, viral, protein, proteins, vpr, replication, virus, vif, human, cells, cellular, cell, host, cycle, vpu, nuclear, interaction, infectivity, factor
**75**	3	3	vaccine, responses, vaccines, immune, vaccination, response, immunization, dna, antibody, immunity, mice, recombinant, induced, immunized, antigens, influenza, immunogenicity, mucosal, cellular, humoral
**76**	3	3	responses, ctl, class, epitopes, response, hla, peptides, t-cell, cytotoxic, cell, mhc, hiv-specific, peptide, epitope, cd8+, specific, viral, immune, control, gag
**77**	2	2	sex, women, sexual, condom, partners, condoms, partner, female, workers, risk, hiv, men, reported, male, sexually, couples, clients, transmitted, transmission, prevention
**78**	3	3	hiv-1, infection, hiv-2, infected, type, hiv-1-infected, virus, human, immunodeficiency, individuals, primary, study, hiv-1-positive, findings, hiv-1-seropositive, uninfected, early, anti-hiv-1, dual, seronegative
**79**	1	1	patients, factor, anemia, severe, complications, platelet, infection, hiv, surgery, hemophilia, surgical, thrombocytopenia, treatment, iron, anaemia, haemophilia, associated, bleeding, patient, risk
**80**	2	2	women, transmission, pregnant, pregnancy, infants, maternal, mothers, hiv, delivery, infant, birth, risk, mother, vertical, mother-to-child, perinatal, breastfeeding, child, milk, breast
**81**	1	1	tissue, lymph, cells, cases, nodes, lesions, tissues, lymphoid, node, leishmaniasis, biopsy, situ, changes, biopsies, detected, staining, findings, observed, skin, revealed
**82**	1	1	positive, negative, test, samples, hiv, tested, tests, western, antibody, elisa, blot, antibodies, assay, blood, specimens, confirmed, sera, serum, eia, screening
**83**	2	2	depression, symptoms, life, health, mental, quality, physical, scores, psychiatric, hiv, psychological, associated, cognitive, study, disorders, scale, functioning, depressive, measures, illness

**84**	2	1	blood, donors, transfusion, donor, hiv, risk, screening, virus, hepatitis, units, products, donations, transmission, infections, safety, donation, recipients, viruses, plasma, transfusions
**85**	3	3	antibodies, antibody, antigen, p24, igg, serum, sera, human, antigens, detected, virus, htlv-i, specific, presence, iga, igm, elisa, monoclonal, titers, assay
**86**	1	1	disease, diseases, clinical, patients, infection, infections, syndrome, manifestations, common, skin, disorders, associated, hiv, diagnosis, conditions, inflammatory, infectious, chronic, reactions, cases
**87**	2	2	cases, aids, hiv, epidemic, transmission, countries, reported, population, infection, europe, spread, number, surveillance, infected, people, china, infections, country, heterosexual, thailand
**88**	3	3	apoptosis, cells, cell, protein, activation, expression, effects, induced, death, kinase, signaling, pathway, role, gp120, stress, increased, human, receptor, activity, pathways
**89**	2	2	hiv, infection, infected, individuals, hiv-infected, persons, transmission, primary, early, increased, infections, acute, uninfected, high, evidence, increase, studies, number, suggests, associated
**90**	1	1	patients, infections, isolates, candida, fluconazole, species, bacterial, infection, albicans, isolated, fungal, meningitis, strains, cryptococcal, antifungal, bacteria, clinical, neoformans, pneumococcal, candidiasis
**91**	1	1	pain, patients, imaging, ct, neuropathy, ocular, peripheral, findings, hiv, retinal, lesions, magnetic, examination, visual, nerve, mri, resonance, eye, loss, clinical
**92**	3	3	role, studies, evidence, factors, data, play, findings, association, effects, well, mechanisms, influence, differences, potential, direct, remains, support, provide, hypothesis, suggested
**93**	2	2	art, adherence, antiretroviral, treatment, therapy, medication, patients, arv, initiation, monitoring, outcomes, medications, patient, settings, regimen, receiving, regimens, failure, hiv, clinical
**94**	1	1	patients, treatment, months, therapy, treated, response, days, median, follow-up, received, three, weeks, patient, time, duration, survival, range, complete, outcome, period
**95**	3	3	tat, transcription, hiv-1, expression, protein, gene, ltr, binding, nf-, rna, promoter, activation, human, tar, activity, cells, terminal, transcriptional, long, repeat
**96**	2	2	hiv, testing, test, screening, tested, counseling, counselling, voluntary, routine, positive, vct, clinic, tests, clinics, rapid, offered, uptake, care, clients, services
**97**	2	2	knowledge, hiv/aids, aids, students, education, attitudes, study, respondents, survey, questionnaire, level, school, awareness, educational, health, high, people, sample, attitude, conducted
**98**	3	3	virus, siv, immunodeficiency, infection, macaques, human, animals, simian, infected, rhesus, monkeys, viruses, model, humans, viral, fiv, animal, macaque, species, shiv

**99**	1	1	virus, immunodeficiency, human, hiv, acquired, syndrome, aids, infection, type, infected, deficiency, immune, -infected, virus-infected, agent, -positive, persons, report, -related, virus/acquired
**100**	3	3	gene, cells, expression, vector, vectors, genes, cell, human, protein, expressed, lentiviral, virus, recombinant, system, transduction, expressing, transfer, transduced, lines, hiv-1

Robustness check of clusters under variability of data

In order to check for the robustness of our topic clusters under the variability of input data (i.e. abstracts), we randomly sliced our data set of abstracts into three slices containing 70064, 69685, and 69859 abstracts. Then, for each category, we followed the procedure of topic modeling, creating the networks of topics, and clustering the topics. In this procedure, we used the same parameters as the original analysis, the only difference being the input data. The clustering algorithm, provided us with three clusters for each slice of data. In order to match the clusters for each slice of data with clusters in the original analysis, we took the top 10 words of each topic and found the set of unique top words for each cluster. Afterwards, we calculated the following similarity index for each pair of clusters from each slice and the original clusters.

$$r_{ij} = \frac{S_{ij}^2}{N_i \hat{N}_j}$$

In the equation above, r_{ij} is the similarity of cluster *i* from the original analysis and cluster *j* of the slice k (where *i*, *j*, and *k* = {1, 2, 3}). Also, N_i is the number of items in the set of unique top words for cluster *i* of the original analysis and $\hat{N}_j$ is the number of items in the set of unique top words for cluster *j* of the slice *k*. Number of shared words in these two sets are captured by S_{ij}. Comparison of the clusters for three slices of data is shown in Table S2. We find that for each slice, any of the clusters is highly similar to only one of the clusters from the original analysis. Hence, the clusters of topics used in this paper as the main item of analysis is robust to the variation in input data.

Table S2.
Comparison of clusters for three slices of data and the original analysis. The table shows the number of shared unique words in the set of unique top words (S_{ij}). The r_{ij} (normalized number of shared unique words) values are reported in parentheses. The gray cells show the corresponding cluster for each slice to the one of the original analysis based on the values of r_{ij}.

		Slice 1			Slice 2			Slice 3		
	Cluster:	1	2	3	1	2	3	1	2	3
	$\hat{N}$:	*230*	*228*	*227*	*258*	*263*	*221*	*268*	*236*	*244*
**Original Analysis**	Cluster 1 (*N = 242*)	179 (0.58)	44 (0.03)	51 (0.05)	195 (0.61)	45 (0.03)	34 (0.02)	174 (0.47)	48 (0.04)	57 (0.05)
	Cluster 2 (*N = 231*)	47 (0.04)	33 (0.02)	174 (0.58)	59 (0.06)	192 (0.61)	24 (0.01)	53 (0.04)	185 (0.63)	28 (0.01)
	Cluster 3 (*N = 206*)	34 (0.02)	179 (0.68)	19 (0.01)	59 (0.06)	22 (0.01)	146 (0.47)	51 (0.05)	22 (0.01)	151 (0.45)

Robustness check of regression results to clustering

In Table S3, we report results of a regression analysis based on the clusters generated by considering the top 11 topics in each paper. Our results hold under new clusters.

Table S3.
Panel data regression results for BSS topics generated by top 11 topics per paper.

		**Dependent Variable:** Percentage of BSS topics in HIV/AIDS Research
**Independent Variables**	Log(HIV/AIDS Mortality)	0.016^{**} (0.003)
	Log(GDP per Capita)	-0.027^{**} (0.005)
	Intercept	0.679^{**} (0.009)
	$F(2,83)$	12042.55^{***}
	R^2	0.281

* p < 0.05 ** p < 0.001 *** p < 0.0001
Standard errors are presented in parentheses.

Regression estimates by using alternative models

We have explained in the manuscript why we have selected the Driscoll-Kraay model as our main analysis tool. Here, in Table S4, we present the results of regression analysis with two other widely used methods: the White-Huber (Model 3) and Newey-West (Model 4) standard errors. As it can be seen, our results holds in both models.

Table S4.

Panel data regression results for percentage of BSS topics.

		**Dependent Variable:** Percentage of BSS topics in HIV/AIDS Research	
		Model 3	Model 4
**Independent Variables**	Log(HIV/AIDS Mortality)	0.016** (0.003)	0.016** (0.004)
	Log(GDP per Capita)	-0.012* (0.006)	-0.027** (0.005)
	Intercept	0.554** (0.054)	0.679** (0.041)
	$F(2, 237)$		50.6***
	$Wald\ \chi^2$	32.73***	

* p < 0.05 ** p < 0.001 *** p < 0.0001
Standard errors are presented in parentheses.

Regression estimates when controlling for time and its interactions

In Table S5, we report the results of our regression analysis (using Huber-White standard errors) including time periods, and the interactions of time period and the other two variables. As it can be observed, while our findings still hold under this condition, neither of terms with time periods were found to be significant.

Table S5.

Panel data regression results for BSS topics including the time variables.

		**Dependent Variable:** Percentage of BSS topics in HIV/AIDS Research
**Independent Variables**	Log(HIV/AIDS Mortality)	0.013* (0.005)
	Log(GDP per Capita)	-0.033** (0.007)
	Time Period	
	(1996-2000)	0.019 (0.052)
	(2001-2005)	-0.012 (0.061)
	(2006-2010)	0.064 (0.067)
	Time Period X Log(HIV/AIDS Mortality)	
	(1996-2000)	-0.007 (0.006)
	(2001-2005)	-0.003 (0.006)
	(2006-2010)	-0.004 (0.006)
	Time Period X Log(GDP per Capita)	
	(1996-2000)	-0.009 (0.007)
	(2001-2005)	0.000 (0.008)
	(2006-2010)	-0.003 (0.009)
	Intercept	0.685** (0.066)
	$Wald\ \chi^2$	187.25***
	R^2	0.31

* $p < 0.05$ ** $p < 0.001$ *** $p < 0.0001$
Standard errors are presented in parentheses.

Regression estimates for papers without cross-country collaboration

In Table S6, we report the results of regression analysis by only considering papers without cross-country collaboration. As shown in Table S6, our findings reported in the main manuscript holds in this subset of data as well.

Table S6.

Panel data regression results for subset of publications without any cross-country collaboration.

		**Dependent Variable:** Percentage of BSS topics in HIV/AIDS Research
**Independent Variables**	Log(HIV/AIDS Mortality)	0.016** (0.003)
	Log(GDP per Capita)	-0.026** (0.005)
	Intercept	0.661** (0.025)
	$F(2, 83)$	1755.00***
	R^2	0.2218

* $p < 0.05$ ** $p < 0.001$ *** $p < 0.0001$

Standard errors are presented in parentheses.
